# Discovering Modality of Cognitive Function Using Clustering Analysis

**DOI:** 10.1093/geroni/igab046.2225

**Published:** 2021-12-17

**Authors:** Stacy Anderson, Thomas Perls, Marianne Nygaard, Paola Sebastiani, Qingyan Xiang

**Affiliations:** 1 Boston University School of Medicine, Boston, Massachusetts, United States; 2 Boston University, Boston, Massachusetts, United States; 3 Department of Public Health, University of Southern Denmark, Odense, Syddanmark, Denmark; 4 Tufts Medical Center, Physician Organization, BOSTON, Massachusetts, United States

## Abstract

In this study with Long Life Family Study (LLFS) participants, we aimed to identify patterns of performance on cognitive function assessments as specific cognitive signatures. We hypothesize that such signatures can be correlated with biomarkers and clinical outcomes. More than 4,700 LLFS participants were administered, at enrollment, a series of neuropsychological tests that measure various cognitive domains. We performed a cluster analysis to group LLFS subjects into clusters characterized by combinations of six neuropsychological test scores. The analysis resulted in 10 clusters of varying size with different cognitive signatures that (1) significantly correlated with physical and pulmonary function, and 31 blood biomarkers and (2) predicted mortality and incident medical events such as dementia, cardiovascular diseases, etc. We conclude that cluster analysis of multiple neuropsychological tests discovers cognitive signatures that are more specific than individual cognitive domains and that these can be correlated with blood biomarkers, incident medical outcomes and mortality.

